# Risk factors for ANA positivity in healthy persons

**DOI:** 10.1186/ar3271

**Published:** 2011-03-02

**Authors:** Quan-Zhen Li, David R Karp, Jiexia Quan, Valerie K Branch, Jinchun Zhou, Yun Lian, Benjamin F Chong, Edward K Wakeland, Nancy J Olsen

**Affiliations:** 1 Department of Immunology, University of Texas Southwestern Medical Center at Dallas, 5323 Harry Hines Blvd., Dallas TX 75390-9093, USA; 2 Department of Medicine, The Division of Rheumatic Diseases, The Simmons Arthritis Center, University of Texas Southwestern Medical Center at Dallas, 5323 Harry Hines Blvd., Dallas TX 75390-8884, USA; 3 Department of Dermatology, University of Texas Southwestern Medical Center at Dallas, 5323 Harry Hines Blvd., Dallas TX 75390-9069, USA; 4 Current address: Division of Rheumatology, Penn State Hershey Medical Center, 500 University Drive, Hershey PA 17033, USA

## Abstract

**Introduction:**

The finding of antinuclear antibody (ANA) positivity in a healthy individual is usually of unknown significance and in most cases is benign. However, a subset of such individuals is at risk for development of autoimmune disease. We examined demographic and immunological features that are associated with ANA positivity in clinically healthy persons to develop insights into when this marker carries risk of progression to lupus.

**Methods:**

Biological samples from healthy individuals and patients with systemic lupus erythematosus (SLE) were obtained from the Dallas Regional Autoimmune Disease Registry (DRADR). Measurements carried out on serum samples included ANA, extractable nuclear antibodies (ENA) and autoantibody profiling using an array with more than 100 specificities. Whole blood RNA samples from a subset of individuals were used to analyze gene expression on the Illumina platform. Data were analyzed for associations of high ANA levels with demographic features, the presence of other autoantibodies and with gene expression profiles.

**Results:**

Overall, ANA levels are significantly higher in females than in males and this association holds in patients with the autoimmune diseases lupus and rheumatoid arthritis (RA) as well as in healthy controls (HC). Age was not significantly associated with ANA levels and the elevated ANA values could not be explained by higher IgG levels. Another autoantibody, anti- cyclic citrullinated peptide (CCP), did not show gender dimorphism in rheumatoid arthritis (RA) or healthy individuals. The autoantigen array showed significant elevations of other autoantibodies in high ANA HCs. Some of these autoantibodies were directed to antigens in skin and others were related to autoimmune conditions of kidney, thyroid or joints. Gene expression analyses showed a greater prevalence of significantly upregulated genes in HCs with negative ANA values than in those with significant ANA positivity. Genes upregulated in high ANA HCs included a celiac disease autoantigen and some components of the Type I interferon (IFN) gene signature.

**Conclusions:**

Risks for ANA positivity include female gender and organ-specific autoimmunity. Upregulation of skin-specific autoantibodies may indicate that early events in the break of tolerance take place in cutaneous structures. Some of these changes may be mediated by Type I IFN. Blood profiling for expressed autoantibodies and genes has the potential to identify individuals at risk for development of autoimmune diseases including lupus.

## Introduction

Antinuclear antibodies (ANAs) are measurable in approximately 25% of the population, and the prevalence of significantly elevated levels may be 2.5% [1]. Findings from numerous studies show remarkable consistency across ethnically and racially diverse study populations despite the use of many different methods for ANA measurement. The persistence of this type of autoreactivity in the human population suggests that antinuclear antibodies may be an important component of the normal immune response. Most individuals with a positive ANA do not have an autoimmune disease and most also are unlikely to develop one. This is consistent with the fact that the prevalence of all autoimmune disorders is 5 to 7% [2]. Furthermore, the disease that is most closely linked to ANA positivity, systemic lupus erythematosus (SLE), is relatively rare, affecting no more than 1 to 1.5 per 1,000 persons (0.1 to 0.15%) in the United States [3]. Nevertheless, since ANA positivity is for all practical purposes a requirement for SLE diagnosis, it must also be assumed that individuals who are in preclinical disease stages are represented in the ANA positive healthy population. Although many consultations for ANA positivity seen in rheumatology practice are not associated with any identifiable pathology, it is also true that if early detection of SLE is to become feasible, focus on the ANA positive population will be necessary.

We have considered the possibility that other blood markers could be used to differentiate benign ANA positivity from that which carries a high risk of autoimmune disease. These markers may include other autoantibodies, since it is well-known that autoantibody positivity increases in quantity and complexity in years preceding a diagnosis of SLE [4]. Gene dysregulation in peripheral blood cells has been closely associated with SLE diagnosis and disease status, so changes in gene expression may also signal a condition with enhanced risk.

To address these questions, we studied healthy individuals and patients with autoimmune diseases who had been enrolled in the Dallas Regional Autoimmune Disease Registry (DRADR). A subgroup of healthy controls that were found to have high ANA levels was examined in greater detail using autoantigen and gene expression arrays. The findings suggest the feasibility of identifying risk markers for development of SLE in the setting of ANA positivity, using both demographic features and profiling of autoantibodies and expressed genes in peripheral blood.

## Materials and methods

### Patients and healthy controls

Samples for study were obtained from the Dallas Regional Autoimmune Disease Registry (DRADR). Individuals are classified according to clinical diagnosis or healthy condition at the time of enrollment into the registry. Disease features and the presence of SLE criteria were determined by patient interview and medical record review [5]. Blood samples were obtained for banking of serum aliquots and whole blood samples were stored in PaxGene tubes for later isolation of RNA. All samples were maintained at -80°C until use. The overall study group included 1,159 individuals from DRADR: 401 healthy controls (HC) who were negative for current or past autoimmune disease, 116 first-degree relatives (FDR), 294 patients with SLE, 151 patients with less than 4 SLE criteria and considered as having incomplete lupus (ILE), 154 with rheumatoid arthritis (RA) and 43 with other miscellaneous conditions including scleroderma, Sjogren's syndrome, ankylosing spondylitis and vasculitis. More detailed analyses were carried out on a subset of HC individuals with high ANA values (*n *= 18) and these were compared to gender- and age-matched HC with negative ANA values (*n *= 16) and to SLE patients with high ANA levels of >100 E.U. (*n *= 14). In one experiment, plasma samples from the Dallas Heart Study population, which has been described previously [1], were employed. All subjects gave written informed consent for entry into the Dallas Regional Autoimmune Disease Registry. Research carried out under the auspices of this registry has been approved by the UT Southwestern Institutional Review Board.

### Antibody assays

Serum ANA levels were measured by ELISA (Inova, San Diego, CA, USA) using the manufacturer's suggested cut-off of >20 units to define positive results. Other ELISA kits were used to measure CCP antibodies (Inova), thyroglobulin autoantibodies (Genway Biotech Inc., San Diego, CA, USA) and total IgG (Bethyl Laboratories Inc., Montgomery, TX, USA). The extractable nuclear antibody (ENA) panel for eight additional specificities was a Luminex-based multiplex assay, and positive results were as defined by the manufacturer (Inova). Serum reactivity to a panel of approximately 101 autoantigens and 6 controls was measured on a slide-based array that has been described previously (Additional file 1) [6]. Serum samples (1 μL, diluted 1:100) were added to each array in duplicate and autoantibodies were detected with Cy3-labelled anti-human IgG and Cy-5 labeled anti-human IgM simultaneously. Images were generated for analysis and mean fluorescence intensities (MFI) were determined as previously described. Heat maps were generated using Cluster and Treeview software (Michael Eisen, Berkeley CA, USA) [7]. On the heat map, intensities higher than the row mean are colored red, those below the mean are green and cells with signals close to the mean are black. Gray was used to denote missing data.

### Gene expression analysis

Total RNA was prepared from 2.5 to 5.0 ml of blood collected in Paxgene tubes that had been stored at -80°C. Purity and concentration of the isolated RNA was determined using Bioanalyzer 2100 (Agilent Technologies, Santa Clara, CA, USA) and Nanodrop 1000 spectrophotometer (Thermo Scientific, Wilmington, DE, USA). We used 250 ng of total RNA to generate biotinylated cRNA using a TotalPrep RNA Amplification kit (Ambion, Austin, TX, USA). The samples were applied to Ilumina HumanWG-6 v3.0 Expression Bead Chips (Illlumina Inc., San Diego, CA, USA) following the manufacturer's directions. The resulting array data were analyzed using Ilumina GenomeStudio software (version 3) and statistical analyses were carried out using Partek Genomic Suite (version 6; Partek Inc., Gladstone, MI, USA). Heat maps were generated using the Cluster and Treeview programs [7].

### Statistical analyses

Data are presented as mean values and standard errors of the mean. Continuous variables in experiments with three or more groups of samples were analyzed using a one-way ANOVA with Tukey's multiple comparison test for post test analysis of pairs of samples or the Kruskal-Wallis test for data that did not fit a normal distribution. Analyses of experimental data with two comparison groups used an unpaired 2-tailed Student's *t*-test, with Welch's correction for groups with unequal variances or Mann-Whitney U for data that did not fit a normal distribution. Pearson's correlation coefficient was used to compare continuous variables. Discontinuous variables were compared using Fisher's Exact Test. Graph pad PRISM software (version 5.0a; GraphPad Software, La Jolla, CA, USA) was used for data analysis and graphics. *P*-values <0.05 were considered significant.

## Results

### ANA and autoantibodies

**Figure 1:**
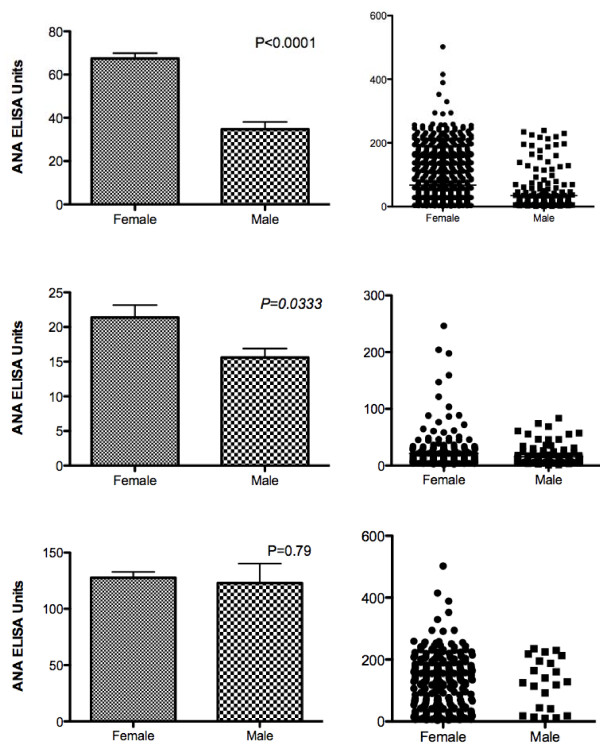
**Serum ANA levels measured by ELISA in individuals enrolled in DRADR**. Results are shown for all individuals, regardless of diagnosis (top panel), for healthy control (HC) subjects only (middle panel) and for SLE patients only (bottom panel). For each set of results, mean and SEM values are shown on the left and individual values are plotted on the right; male and female groups are compared using *t*-tests. ELISA values greater than 20 units are considered positive.

Using the defined cut-off of >20 ELISA units (EU), 615 individuals out of the 1,159 tested were ANA positive. For the subset of 401 HC, the average ANA was 19.5 EU and 101 individuals were in the positive range. This rate of 25% HC positivity is very close to what we have reported previously [1]. Healthy FDRs had a slightly higher overall average ANA (24.4 EU) and a prevalence of ANA positivity of 34%. These values were not significantly different than in the HCs (*P *>0.07 for both). For the overall group, ANA values were significantly higher in females than in males (Figure 1) and no males had values >240 EU while 3.2% of females had values in this range (*P *= 0.0030). When the analysis was limited to HC, females again showed significantly higher ANA levels than males (21.4 vs. 15.6 EU; *P *= 0.033) and ANA positivity was also more prevalent in HC females (29%) than in HC males (17%; *P *= 0.014). This corresponds to a relative risk of ANA positivity in healthy females vs. males of 1.21 (95% confidence interval = 1.059 to 1.390). The highest ANA levels were seen almost exclusively in non-Hispanic females (data not shown), although the overall mean values for Hispanic and non-Hispanic females were not significantly different (*P *= 0.7). African-American (AA) HC (*n *= 32) had a higher mean ANA value than non-AAs (27.47 ± 5.8 EU vs. 18.1 ± 5.8 EU), but the difference did not reach statistical significance (*P *= 0.064). Within each gender, AA individuals also had higher values than non-AA individuals (females: 30.38 ± 8.12 EU vs. 20.59 ± 1.80 EU; males 21.09 ± 5.55 EU vs. 15.17 ± 1.31 EU); these differences were also not significant (*P *>0.1 for each). However, the results are highly suggestive of higher ANA values in AA HC, and the differences might achieve significance in a larger sample size. Patients with SLE did not show gender differences in overall ANA values, although the very highest ANA values were again seen exclusively in females (Figure 1).

**Figure 2:**
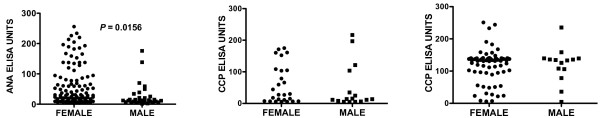
**Serum antibody levels measured by ELISA in RA patients and community-derived control subjects**. ANA levels in RA patients from DRADR (L panel) were significantly higher in females than in males. By contrast, CCP antibodies measured in individuals from the Dallas Heart Study (middle panel) or in DRADR RA patients (right panel) did not show significant male-female differences.

We compared these findings to another autoimmune disease, RA, which is associated with the CCP autoantibody. While RA patients showed significantly higher ANA levels in females than in males (Figure 2), antibodies to CCP did not show gender differences in either the control population represented by the Dallas Heart Study or in RA patients from DRADR (Figure 2). These findings suggest that female gender is a general risk factor for ANA but is not necessarily a correlate with other autoimmune disease-specific antibodies.

Age was not related to ANA positivity in HCs; high values were scattered throughout the age spectrum (R^2 ^= 0.01). The high ANA levels in HC also were not explained by overall increases in total IgG as the two measures were not significantly correlated (R^2 ^= 0.11; *P *= 0.2).

To further understand high ANA levels in HC, a subgroup analysis was done using HC subjects with ANA values greater than 40 EU as the index group. This value is approximately one standard deviation greater than the overall mean ANA for HC. A total of 18 of the 401 HC, or about 4%, fit this definition of high ANA. Two comparator groups, HC with negative ANA (ANA values <12 EU) and SLE patients with high ANA (>100 EU) were generally matched for demographic features including gender, race and ethnicity (Table 1). Autoantibodies on the ENA panel were generally not elevated in the high ANA HC group. Only 3 of the 18 individuals showed positive ENA results, and in all three the lone positive specificity was anti-chromatin.

**Table 1:** Demographic features of study groups

Group	Age (yrs)	ANA (EU)	Female (%)	AA (%)	Hispanic (%)
HC High ANA (*n *= 18)	33.8 ± 3.4*	78.4 ± 10.0*	*83*	*22*	6
HC Low ANA (*n *= 16)	43.1 ± 3.7	7.0 ± 0.6	*75*	*25*	0
SLE (*n *= 14)	38.0 ± 4.0	182.4 ± 15.4	*93*	*43*	7
P**	0.2	0.001^#^	0.4	0.4	0.6

*Values represent mean and standard error of the mean. **P-values were calculated by one-way ANOVA or Fisher's exact test. ^# ^Mean ANA in SLE was significantly higher than in the High ANA HC group (*P *<0.05). ANA, antinuclear antibodies; SLE, systemic lupus erythematosus.

**Figure 3:**
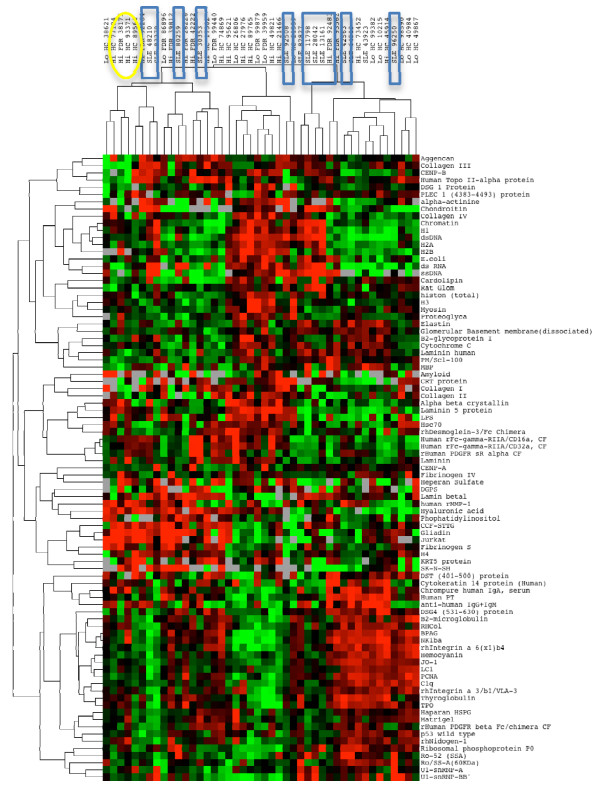
**Heat map with clustering of IgM autoantibodies HC subjects and SLE patients**. Clusters of study subjects are highlighted to show groups of SLE patients (blue boxes) and ANA high HC (yellow ovals).

**Figure 4:**
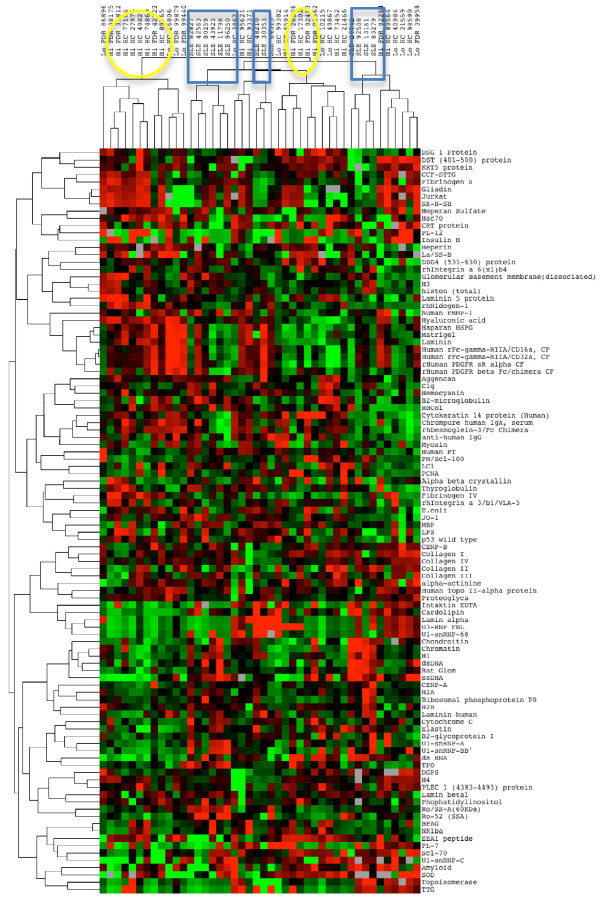
**Heat map with clustering of IgG autoantibodies in HC subjects and SLE patients**. Clusters of study subjects show groups of SLE patients (blue boxes) and ANA high HC (yellow ovals).

The three study groups were then compared using the autoantigen array for both IgG and IgM autoantibodies (Figures 3 and 4). Two major IgM clusters showed a tendency for the SLE patients to cluster together, but this was not statistically significant (*P *= 0.08); the high ANA HC individuals had a similar tendency to be in the non-SLE cluster (*P *= 0.06; Figure 3). For IgG, two primary IgG clusters were identified, and all of the SLE patients were in one of these clusters (*P *= 0.009; Figure 4).

**Figure 5:**
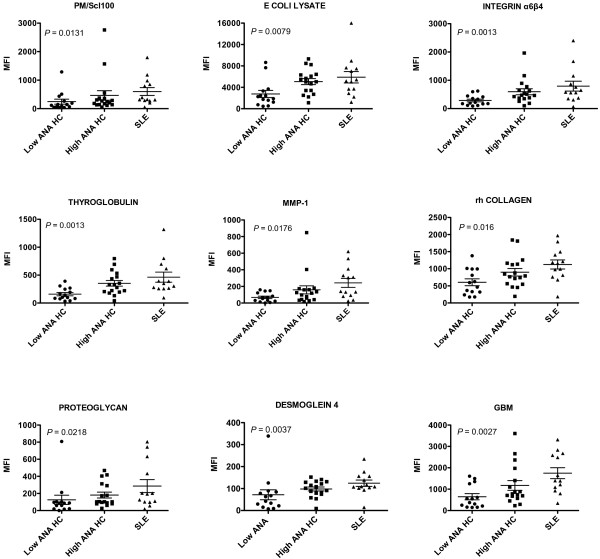
**IgG autoantibodies showing significant stepwise elevations in the three study groups**. *P-*values calculated using Kruskall-Wallis test.

**Figure 6:**
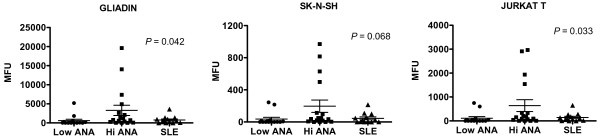
**IgG autoantibodies with elevated levels in high ANA HCs but not in SLE patients**. IgM reactivity for these same three autoantigens had similar patterns (not shown).

Antibodies detected on the arrays were then examined for specificities appearing in HC that were lupus-like. This was defined as overall mean values for a given specificity showing no difference between the high ANA HC and SLE groups (*P *>0.1) while at the same time having lower values in the low ANA HC group compared to SLE (*P *<0.1). The antibodies in these groups were examined to choose those with stepwise increases in the groups (low ANA HC <High ANA HC <SLE) and to exclude any with distributions that were highly skewed by one or two individuals. This analysis yielded 14 IgGs; 9 of these with the most significant *P*-values (Kruskal-Wallis test) are shown (Figure 5). Four of the nine specificities are directed against autoantigens found in skin tissues (DSG4, MMP1, recombinant human collagen, integrinα6β4) [8-10]. Others are associated with autoimmune kidney disease (GBM), thyroid disorders (thyroglobulin), scleroderma (PM/Scl100), and inflammatory arthritis (proteoglycan) [11]. A search for autoantibodies that might be associated with a lowered risk of lupus was carried out by choosing specificities that were elevated in high ANA HC but were not high in SLE patients. The ratio of the mean values for the high ANA HC group to the corresponding SLE group mean was calculated, and three specificities had IgG ratios greater than 4.0: Jurkat T cells, gliadin and SK-N-SH (Figure 6). In each of these, a subset of the high ANA HC individuals showed strong reactivity while SLE patients did not have high values. For gliadin, an autoantigen associated with celiac disease, and Jurkat, a measure of anti-T cell and thymocyte antibodies, the distributions were significantly different for the three groups (*P *= 0.042 and 0.033, respectively). Although the antineuronal antibodies measured by SKNSH reactivity did not show significant differences across groups by ANOVA, a dichotomized analysis showed values greater than 400 MFI units were present only in the high ANA HC group (*P *= 0.0242). The four highest values for each of these different autoantibodies represented the same individuals.

An independent ELISA assay was carried out for thyroglobulin (TG) autoantibodies to compare with the findings on the array. Anti-TG levels of the IgG class measured by the two techniques were significantly correlated (*P *= 0.007) and values were significantly higher in the HC high ANA group (1.18 ± 0.23 EU) than the HC low ANA group (0.60 ± 0.07 EU; *P *= 0.030), consistent with the array results.

### Gene expression profiles

Mean gene expression values were compared between the two HC groups defined by ANA status, and this analysis identified 95 dysregulated genes at a significance level of *P *<0.01. Somewhat surprisingly, the vast majority of these genes (90 out of 95) were upregulated in the ANA-negative group. Of the five genes that were upregulated in the high ANA HC group, the highest (two-fold difference) was *TGM2*, which encodes the celiac disease autoantigen transglutaminase 2.

**Figure 7:**
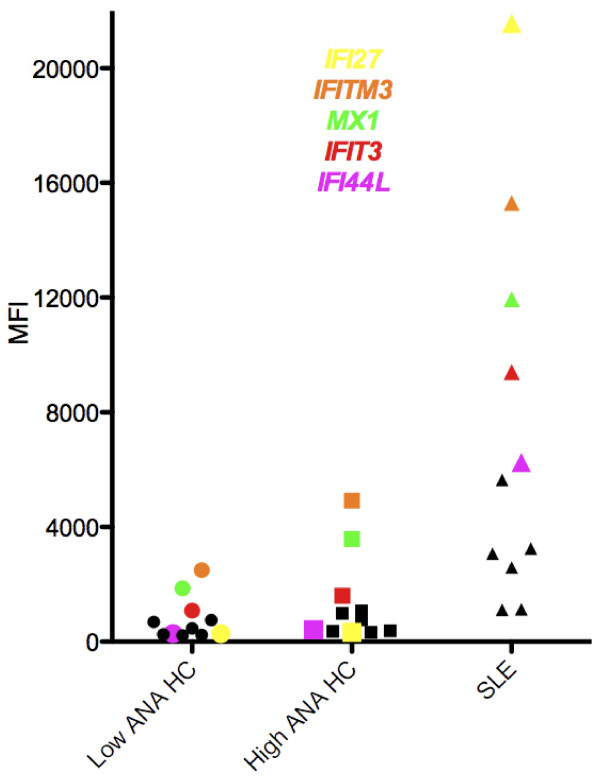
**Expression of Type I IFN signature genes in the three study groups**. A stepwise pattern of increase is observed for *IFITM3, MX1 *and *IFIT3*. The genes *IFI44L *and *IFI27 *show increases that are relatively specific for SLE. Other IFN genes without elevation in the high ANA HC group shown with black symbols are: *IFI6, HES4, ISG15, OAS1, IFIT3, HERC5, EIF2AK2*.

To detect specificities that might be informative of ANA and diagnosis status, a second analysis was carried out by first comparing the SLE and high ANA HC groups to determine gene specificities that were significantly different at a significance level of *P *<0.001. This list was then sorted based on the values for the calculated ratio SLE/ANA-High HC. The most highly expressed specificity in this list was *IFI27*, which was 65-fold higher in SLE than in ANA High HC. Overall, 69 genes in this list had greater than two-fold differences between SLE and ANA High HC. These 69 genes were then resorted based on differences in the ratio of ANA High HC/ANA Low HC. The highest ratio in this set was two-fold and out of the top 10 specificities, 7 were in the Type I interferon signature (*IFITM3, MX1, IFI6, IFI44L, ISG15, OAS1, IFIT3*) and one encodes a protein that is activated by dsRNA as might be present in a viral infection (*EIF2AK2*). The specificities that most clearly showed stepwise increases in the three groups going from Low ANA HC to High ANA HC to SLE were *IFITM3 *and *MX-1 *(Figure 7).

## Discussion

Detecting autoimmune disease in early or preclinical stages is clinically important because the institution of treatment prior to the onset of organ damage has a greater chance to ameliorate or even cure the disease [12]. However, early and reliable diagnosis of lupus is a challenge, in large part due to the performance profiles of available diagnostic tests. The optimal test would be sensitive enough to detect all individuals who have a disease while at the same time delivering sufficient specificity to have reasonable predictive probability that the disease is likely. The classic screening test for SLE is the presence in serum of antinuclear antibodies (ANAs) measured by immunofluorescence staining of a cellular substrate [13]. ANA positivity is for all practical purposes required to make a diagnosis of lupus since more than 99% of patients with SLE have significant levels of this autoantibody detected at some time during the course of disease. However, since the prevalence of SLE is low, most individuals presenting to a physician with ANA positivity do not in fact have lupus and are not at high risk for developing this disease. But there are few available quantitative and objective measures to establish prognosis for an individual with a positive ANA. This contrasts to the tools that are available for determining the risk of cardiovascular disease, where multifaceted profiles including elements of personal and family history, social habits, body measurements and lab tests can generate a reasonable and very personalized risk prediction for an individual patient [14].

In practice, physicians actually do employ some profiling to assess the risk associated with ANA positivity. Young women are more likely to develop lupus than old men, for example, so ANA positivity in the former is of greater concern. The present study confirms our previous observation that female gender is a risk factor for significant ANA positivity [1]. This result is also consistent with other findings in healthy control populations including a study of 500 normal individuals in Brazil showing that ANA positivity was almost twice as prevalent in females as in males [15]. Similar findings were reported in a rural Canadian population, with the gender difference being greatest at higher ANA levels, as was also noted in the present study [16]. The enhanced female risk profile does not appear to extend to the anti-CCP antibody that is associated with RA, another female-predominant autoimmune disorder. Reasons for the association of female gender with strong ANA positivity remain obscure.

Age was not correlated with ANA levels in HCs, which seems to contradict the generally-accepted hypothesis that immunosenescence is associated with increased autoantibody production due to decreased self-regulatory mechanisms. The present findings are consistent, however, with other reports [15] and suggest that in a cross-sectional analysis such as this there are many reasons for ANA positivity. The younger individuals may in fact have abnormal immune regulation that predisposes to SLE-like disease while older persons may develop autoreactivity as part of aging immune responses that do not lead to development of pathology.

Other clues are available to suggest approaches to stratifying risk in the ANA-positive population. One is the well-recognized presence of other autoantibodies that accumulate prior to SLE diagnosis [4]. The prevalence of any one of these is low, however. For example, ribosomal P autoantibodies are highly specific for SLE but are present in less than one-third of patients [17]. This finding predicts that if autoantibodies are included in risk profiling, multiplexed assays will be required. One such approach that is available clinically, the ENA panel, was not useful in the high ANA HC individuals in the present study because very few positives were found. This result is consistent with our previous experience indicating that only the high ANA positives are likely to have ENA specificities [1] and even then, the prevalence of other autoantibodies in HCs is very low. The alternative approach which has been applied in the present study is to greatly expand the repertoire of autoantibodies that can be probed by use of an autoantigen array, which in other studies has been shown to provide novel insights into expressed autoantibody repertoires [18]. The array data in the present study revealed increased autoreactivity in a group of high ANA HCs. One of the elevated autoantibodies was thyroglobulin, and since autoimmune thyroid disease is probably about 10-fold more common than SLE [19], this result suggests that a significant proportion of the ANA positivity seen by rheumatologists is related to thyroid autoimmunity. Longitudinal studies suggest that thyroid autoreactivity, especially in women, may be predictive of thyroid dysfunction [20]. Autoantibodies to cartilage proteoglycan can be measured in several systemic and joint-specific rheumatic diseases including Sjogren's Syndrome, rheumatoid arthritis, lupus and ankylosing spondylitis [21], suggesting that undetected or preclinical joint inflammation may contribute to ANA positivity. The relative increase in skin autoreactivity in the high ANA HC group might be related to a relative enrichment for skin antigens on the arrays, so this finding should be interpreted with caution. However, it does raise the interesting question of whether some early autoimmune events might take place in cutaneous structures. Transient ANA positivity has been observed in patients with polymorphous light eruption [22] and exposure to sun in susceptible individuals can trigger major organ-damaging lupus [23]. The interface dermatitis that characterizes the pathology in SLE as well as in other skin disorders may precede a diagnosis of lupus [24] and is associated with activation of the Type I interferon gene signature [25]. The triggering of this set of genes in skin, however, is not limited to inflammatory disease but can also occur as part of the immune response to viruses, raising the question of whether cutaneous reactions to environmental agents in susceptible hosts might generalize to a systemic response.

The autoantibody arrays show that although the autoreactivity spectrum in SLE is broad, not all specificities are elevated. Upregulated autoantibodies to gliadin, and to T lymphocytes and neuroblastoma cells were present only in the ANA high HC group, and not in the SLE patients. This result confirms in part our previous report that gliadin autoreactivity is associated with incomplete forms of lupus that are associated with myopathies [26]. Whether these autoantibodies are actively protective and lower the risk of lupus or alternatively are predictive of other autoimmune diseases developing in these individuals will require further longitudinal investigation.

Upregulated genes observed in high ANA HC individuals include some in the Type I IFN signature that are associated with SLE [27,28]. While some of these genes, notably *IFI27*, were only elevated in SLE, others such as *MX-1 *showed an intermediate level in the High ANA HC group. In addition to being the most highly-upregulated of the IFN genes in our sample, other data suggest that *IFI27 *is relatively more specific for lupus than at least some of the other IFN-inducible genes. A recent study demonstrated that *IFI27 *is more likely to be upregulated in lupus than in another autoimmune condition, idiopathic thrombocytopenic purpura [29]. The specific functions of many of the proteins associated with IFN-related genes are obscure, but *MX-1 *is closely associated with the response to the influenza virus, so upregulation of this gene in normal individuals might reflect the ubiquitous exposure to this pathogen.

The present data suggest possible components of a lupus risk profile. As in the cardiovascular risk profiling equations, gender will be a factor but in lupus the risk will be associated with females rather than males. The age category will be inverted from that of cardiovascular disease, with greater weight given to younger ages. The lupus risk is correlated with ANA levels, not just positivity, with values in the upper quartile having a two- to three-fold elevation of risk [1], so this will be an important component. Other autoantibodies that may add to risk include those that are clinically well-known like anti-dsDNA and anti-Sm, as well as other novel specificities including the skin determinants identified in the present study. On the other hand, antineuronal, anti-thyroid or gliadin autoantibodies might steer attention away from SLE towards other autoimmune disorders. Elevated expression of genes related to the Type I IFN signature is likely to add points to the risk equation.

This study has several limitations. One is the lack of information regarding use of medications, especially hormones, by the HC. Whether administration of estrogen in the form of oral contraceptives or postmenopausal replacement therapy might induce high ANA levels in a healthy individual cannot be ascertained from our data. Another limitation is the cross-sectional design which does not permit insights into changes that evolve over time. And it would be of interest to determine reactivity to foreign antigens such as infectious agents to further interpret the significance of the autoreactive responses. Ultimately, validation of risk profiles will require longitudinal studies.

Finally, since SLE is a relatively rare disorder, the probability of finding a new onset patient is low even after ANA positivity has been identified. One approach to increasing the likelihood of useful results would be to follow individuals who already have some of the identified risk profile components. For example, studies could be carried out in individuals who have been sent for ANA testing for any reason. The very fact that the individual sought medical attention and that an ANA was ordered is likely to increase the pre-test probability of disease. Such an approach has shown that in the population of individuals sent for rheumatoid factor testing, the pre-test probability for RA is 17% [30].

## Conclusions

This study shows that it may be possible to identify the small percentage of ANA positive individuals who are at risk for development of SLE. Characterization of protein and gene expression profiles accompanying ANA positivity has potential to enable more precise risk quantification and ultimately to identify pre-clinical stages of this disease that would allow early and definitive treatment.

## Abbreviations

ANA: antinuclear antibody; CCP: cyclic citrullinated peptide; DRADR: Dallas Regional Autoimmune Disease Registry; ENA: extractable nuclear antibody; EU: ELISA units; FDR: first degree relative; HC: healthy controls; IFN: interferon; ILE: incomplete lupus; MFI: mean fluorescence intensity; RA: rheumatoid arthritis; SLE: systemic lupus erythematosus; TG: thyroglobulin

## Competing interests

NJO has equity interest in ArthroChip LLC and has received research grants from Medimmune, Novo Nordisk, Genentech/Roche and Human Genome Sciences. DRK has received a research grant from Human Genome Sciences. All other authors declare that they have no competing interests.

## Authors' contributions

QZL supervised the protein and gene expression array assays and analyses. DRK organized the patient registry. JQ organized and performed laboratory assays. JZ performed the protein array assays, while YL performed data analyses for protein and gene expression array assays. VKB coordinated patient recruitment for the registry. BFC contributed to design and validation of the autoantibody array. EKW conceived of the study design and NJO assembled the patient cohorts and supervised clinical phenotyping. QZL, DK, EKW and NJO wrote the manuscript with input and consensus from all authors. All authors read and approved the final manuscript.

## Supplementary Material

Additional file 1 **Components of the Autoantigen Array**. This table lists the components of the array along with sources of the autoantigens. Click here for file
