# Supplementary material for: Risk factors for ANA positivity in healthy persons
Source: Arthritis Res Ther. 2011 Mar 2;13(2):R38. doi: 10.1186/ar3271 (PMC3132017; doi:10.1186/ar3271)
Supplement: Additional file 1 — Components of the Autoantigen Array. This table lists the components of the array along with sources of the autoantigens. [file ar3271-S1.PDF]

Supplementary Table 1: Components of the Autoantigen Array

Name	Vendor	Catalog Number
(HSPG) Heparan sulfate proteoglycan	Sigma	H4777
Aggrecan	Sigma	A1960
alpha-actinin	Sigma	A9776
Amyloid	Sigma	A1075
AN-16000 PM/Scl-100bv	Diarect AG	16000
B2-glycoprotein I (Apolipoprotein H)	Diarect AG	14900
B2-microglobulin	Fitzgerald	RDI-CBL62020
BSA	Sigma	A3059
C1q	Alpha Diagnostic	C1Q15-N
Cardiolipin	Sigma	C0563
CENP-A	Diarect AG	16900
CENP-B	Diarect AG	12500
Chondroitin sulphate	Sigma	C4384
Chromatin	Dr. Mohan	
Collagen I	Chondrex	2015
Collagen II	Chondrex	1005
Collagen III	BD Pharmingen	354244
Collagen IV	BD Pharmingen	354245
Cytochrome C	Sigma	C2037
cytochrome P450 (LKM1)	Diarect AG	13500
DGPS (1,2-diacyl-sn-glycero3-phospho-L-serine)	Sigma	P5660
dsDNA	Sigma	D1501
dsDNA (plasmid)	Diarect AG	16000
Elastin	Sigma	E1625
Fibrinogen I-S	Sigma	F8630
Fibrinogen IV	Sigma	F4753
Gliadin(IgG)	Diarect AG	17200
glomerular Basement membrane	Diarect AG	15900
Glomerular Basement membrane(dissociated)	Diarect AG	16800
Good pasteur	Wieslab	WB1029
Histone H1	Roche	223-549
Histone H2A	Roche	1034740
Histone H2B	Roche	223-514
Histone H3	Roche	1034-758
Histone H4	Roche	223-492
HEL	Dr. Mohan	
Hemocyanin	Sigma	H5654
Heparan Sulfate	Sigma	H4777
Heparin	Sigma	H4784
Histone, total	Roche	223565
Hyaluronic acid	Sigma	H7630
Intaktin EDTA	Dr. Mohan	
Intrinsic factor	Diarect AG	16700
JO-1 human	Sigma	J4144
Ku(p70/p80)	Diarect AG	50144
La/SS-B	Sigma	L-9663
Laminin	Sigma	L2020
LC1 (Formiminotransferase Cyclodeaminase)	Diarect AG	13700
Matrigel	BD Pharmingen	356234
Myosin	Sigma	M1636
OVA2	Dr. Mohan	
PCNA (proliferating cell nuclear antigen)	Diarect AG	15100
Phophatidylinositol	Sigma	P0639
PL-12 (alanyl tRNA Synthetase)	Diarect AG	15700

Supplementary Table 1: Components of the Autoantigen Array

Name	Vendor	Catalog Number
PL-7 (Threonyl tRNA synthetase)	Diarect AG	15600
Proteoglycan	Sigma	P5864
Rat Glomeruli	Dr. Mohan	
Ribosomal phosphoprotein PO	Diarect AG	14100
Ro/SS-A(60KDa)	Diarect AG	155000
Ro-52 (SSA)	Sigma	R8526
Scl-70	Diarect AG	12400
SM/RNP	INOVA Diagnostics	708415
SSA/SSB	INOVA Diagnostics	708455
ssDNA	Dr. Wakeland	
Thyroglobulin (non recombinant)	Diarect AG	12200
Topoisomerase	Sigma	T-9069
TPO	Diarect AG	12100
TTG (tissue transglutaminase)	Diarect AG	15200
U1-snRNP-68	Diarect AG	13000
U1-snRNP-A	Diarect AG	13100
U1-snRNP-BB'	Diarect AG	13300
U1-snRNP-C	Diarect AG	13200
Vimentin	Sigma	V4383
Vitronectin	Sigma	V8379
collagen V (human)	BD Pharmingen	354246
collagen VI	BD Pharmingen	354261
vimentin	BD Pharmingen	550513
MBP (Myelin Basic Protein)	Fitzgerald	RDI-TRK8M79
CNPase		
ds RNA		
yeast tRNA		
CCF-STTG1		
E.coli		
Hep2		
Jurkat		
Sk-N-SH		
MBP (Myelin Basic Protein)		
GFAP		
Galactocerebosides		
LPS		
Insulin in Hepes		
Tetanus		
alpha beta crystallin		
human rMMP-1		
rHuman PDGFR beta Fc/chimera CF		
rHuman PDGFR sR alpha CF		
Human rFc-gamma-RIIA/CD32a, CF		
Human rFc-gamma-RIIA/CD16a, CF		
Human Topo II-alpha protein		
Lamin alpha		
Lamin betal		
p53 wild type		
SOD		
BPAG		
Human PT		
U3-RNP FBL		
Hsc70		

Supplementary Table 1: Components of the Autoantigen Array

Name	Vendor	Catalog Number
rhDesmoglein-3/Fc Chimera	R&D systems	1720-DM
rhNidogen-1	R&D systems	2570-ND
rhIntegrin a 6(x1)b4	R&D systems	5497-Ab
rhIntegrin a 3/b1/VLA-3	R&D systems	2840-A3
Cytokeratin 14 protein (Human)	abcam	ab73637-20
Laminin 5 protein	abcam	ab42326-10
EEA1 peptide	abcam	ab14946-100
DSG 1 Protein	Abnova	H00001828-PO1
DSG4 (531-630) protein	Abnova	H00147409-QO1
PLEC 1 (4383-4493) protein	Abnova	H00005339-qO1
KRT5 protein	Abnova	H00003852-PO1
CRT protein	Abnova	H00001401-PO1
DST (401-500) protein	Abnova	H00000667-QO1
Laminin human	Sigma	L4544
NK1ba		
RHCol		
